# EUGHS NEWS

**DOI:** 10.7189/jogh.05.010204

**Published:** 2015-06

From this issue onwards, we are adding an additional item to our regular News section – *EUGHS News*. This section will regularly review the activities of our thriving student's society – Edinburgh University Global Health Society (EUGHS) – during the previous semester. In this inaugural section, we will start with reviewing the entire track record of student research projects and publications in global health with Professors Harry Campbell and Igor Rudan and Drs Harish Nair, Evropi Theodoratou, Lina Zgaga, Davies Adeloye and Kit Yee Chan in the period 2006–2015. We will also review students' attendance at International Conferences, their presentations at these conferences, and their internships at the World Health Organization's Headquarters in Geneva, Switzerland, which were arranged and supported through research projects of the above group of researchers and the *Journal of Global Health*.

**Figure:**
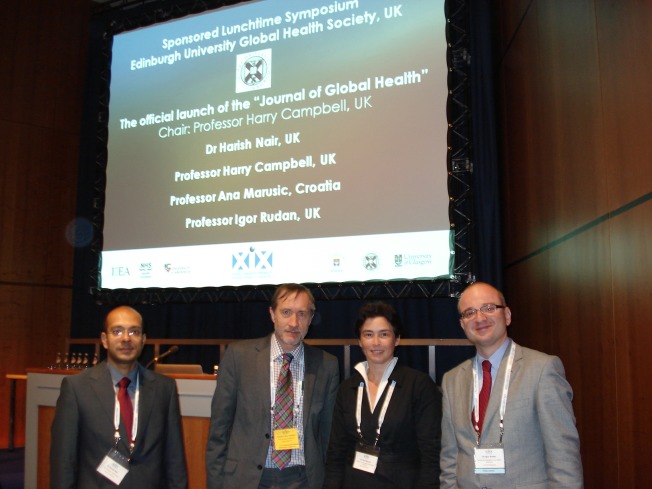
Photo: The launch of the *Journal of Global Health* at the World Congress of Epidemiology in Edinburgh, August 2011. From left: Dr Harish Nair, Professor Harry Campbell, Professor Ana Marušić and Professor Igor Rudan.

Following this documentation of all the activities to date, we are bringing two personal accounts from the two EUGHS interns at the World Health Organization in 2014: Rachel Burge and Katy Wong.

## Track record of EUGHS student research projects, publications, participations at international conferences and internships at the World Health Organization

Professors Harry Campbell and Igor Rudan are Joint Directors of the Centre for Global Health Research at the Usher Institute, the University of Edinburgh; Joint Directors of the World Health Organization's Collaborating Centre in Edinburgh; and Joint Editors–in–Chief, “Journal of Global Health”. With their narrow team of collaborators in global health epidemiology – Drs Harish Nair, Evropi Theodoratou, Lina Zgaga, Davies Adeloye and Kit Yee Chan – they mentored a larger number of undergraduate students towards research projects such as SSC2, SSC4 and BMedSci. These projects were focused on global health themes and they typically involved a systematic review of the literature on a clinical or public health topic in maternal or child health that filled an existing gap in knowledge. There was usually some choice in the topic to suit the interest of the student. The topic was selected at the start of the SSC2, SSC4 or BMedSci to ensure it was topical and sufficiently novel. The work usually involved some interaction with international collaborators, eg, developing country physicians, international health experts or technical officers from the World Health Organization and UNICEF. The project usually contributed to ongoing international research projects of the group and lead to a paper submitted for publication, in which the students were typically lead authors.

**Figure:**
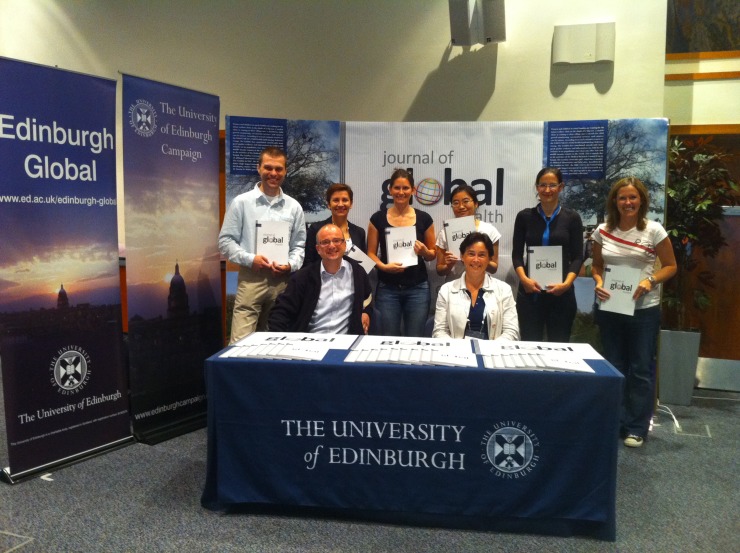
Photo: Editors and EUGHS members at the launch of the *Journal of Global Health* at the World Congress of Epidemiology in Edinburgh, August 2011.

In the period between 2006 and 2015, we managed to publish 57 student publications in international peer–reviewed journals, involving a total of 73 students (as some of their theses contributed to the same publication as specific components). In 38 of these publications (ie, two–thirds), the contribution of the students was substantial enough to justify lead authorship.

Based on these research results, our students took part in 12 international conferences in global health, where they made 34 oral presentations. These meetings were typically organized by the World Health Organization, The Bill and Melinda Gates Foundation or other leading global health institutions. Having a publication and/or a presentation at an international conference has been helping our students to be successful in their applications for placements and jobs across the UK following their graduation.

**Figure:**
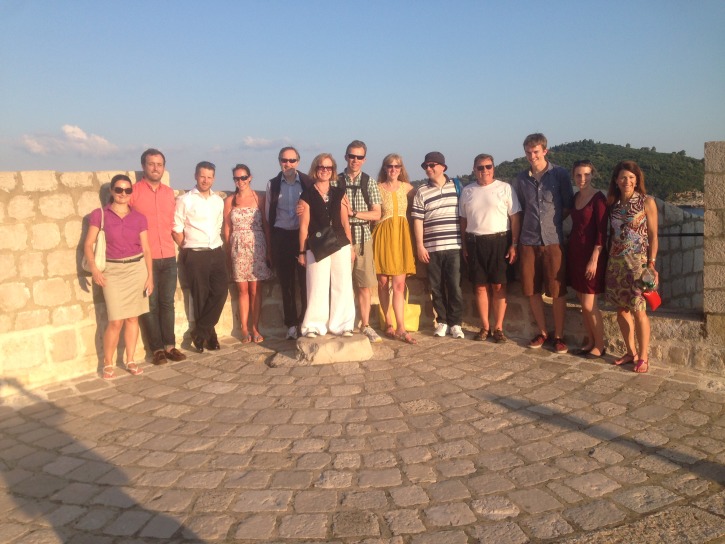
Photo: Several EUGHS members take part at the International Meeting on Maternal and Child Epidemiology Estimates in Dubrovnik, Croatia, in July 2014. Here, photographed with colleagues from the Johns Hopkins University in Baltimore, USA, and London School of Hygiene and Tropical Medicine, UK.

Finally, from 2014 we started arranging internships for EUGHS students at the World Health Organization's Headquarters with our collaborators, which we support through *Journal of Global Health*. Six students took part in these internships to date. A complete lists of activities and students is presented in the following sections.

## SSC2, SSC4 or BMedSci student projects published or accepted for publication 2006–2015

**Table 1** presents a complete list of student publications, to the best of our knowledge (and memory). The name of the contributing student is underlined in each publication.

**Table 1:** List of EUGHS student publications

Authors	Title	Journal
Wijesingha S, Graham S	Evidence behind the WHO guidelines: hospital care for children. What are the clinical indicators of PCP?	J Trop Pediatr 2007;53:4–7
McCallum AD, Duke T	Evidence behind the WHO guidelines: hospital care for children. Is caffeine useful in the prevention of apnoea of prematurity?	J Trop Pediatr. 2007;53:76–7
Bulteel N, Henderson P	Evidence behind the WHO guidelines: hospital care for children. What are the risks of HIV transmission through breast feeding?	J Trop Pediatr. 2007;53:298–302
Bulteel N, Henderson P	Evidence behind the WHO guidelines: hospital care for children. What are the risks of formula feeding in children of HIV–infected mothers?	J Trop Pediatr. 2007;53:370–3
Best J, Hughes S	Evidence behind the WHO guidelines: hospital care for children. What are the useful clinical features of bacterial meningitis found in infants and children?	J Trop Pediatr. 2008;54:83–86
Woodfield G, Dugdale A	Evidence Behind the who guidelines: hospital care for children. What is the most effective antibiotic regime for chronic suppurative otitis media in children?	J Trop Pediatr. 2008;54:151–6
Woodfield J, Re P, Argent A	Evidence behind the WHO guidelines: hospital care for children. what is the most appropriate anti–microbial treatment for tuberculous meningitis?	J Trop Pediatr. 2008;54:220–4
Thurey J, Molyneux E	Evidence behind the WHO guidelines: hospital care for children. The usefulness of Azole prophylaxis against cryptococcal meningitis in HIV–positive children	J Trop Pediatr. 2008;54:361–3
Chandy E, McCarthy J	Evidence behind the WHO guidelines: hospital care for children. What is the most appropriate treatment for giardiasis?	J Trop Pediatr. 2009;55:5–7
Subhi R, Adamson M, Campbell H, Weber M, Smith K, Ashraf H, Berkley J, Bose A, Brent A, Brooks WA, Bruce N, Chisti MJ, Gessner BD, Gyr N, Mwaniki M, Nadjm B, Nokes DJ, Okiro EA, Reyburn H, Sutanto A, Zaman A, Duke T	The burden of hypoxaemia among children in developing countries	Lancet Infect Dis. 2009;9:219–27
Theodoratou E, Johnson S, Jhass A, Madhi SA, Clark A, Boschi–Pinto C, Bhopal S, Rudan I, Campbell H	The effect of *H*aemophilus *influenzae* type b and pneumococcal conjugate vaccines on childhood pneumonia incidence, severe morbidity and mortality.	Int J Epidemiol. 2010;Suppl 1:i172–85
Theodoratou E, Al–Jilaihawi S, Woodward F, Ferguson J, Jhass A, Balliet M, Kolcic I, Duke T, Rudan I, Campbell H	The effect of case management on childhood pneumonia mortality in developing countries	Int J Epidemiol. 2010;Suppl 1:i155–71
Jabeen A, Theodoratou E, Yakoob MY, Eisele TP, Ferguson J, Jhass A, Rudan I, Campbell H, Black RE, Haider BA, Bhutta ZA	Preventive zinc supplementation on mortality due to diarrhoea, pneumonia and malaria	BMC Public Health. 2011;11 Suppl 3:S23
Calder D, Qazi S	Evidence behind the WHO guidelines: hospital care for children. What is the aetiology of pneumonia in HIV–infected children in developing countries?	J Trop Pediatr. 2009;55:219–24
Ford A, Campbell H, Duke T	Pathogens and treatment of chronic diarrhoea in HIV	J Trop Pediatr. 2009;55:349–55
Higginson D, Theodoratou E, Nair H, Huda T, Zgaga L, Jadhav SS, Omer SB, Rudan I, Campbell H	An evaluation of respiratory administration of measles vaccine for prevention of acute lower respiratory infections in children	BMC Public Health. 2011;11 Suppl 3:S31
Nair H, Verma VR, Theodoratou E, Zgaga L, Huda T, Simőes EA, Wright PF, Rudan I, Campbell H	An evaluation of the emerging interventions against respiratory syncytial virus (RSV)–associated acute lower respiratory infections in children	BMC Public Health. 2011;11 Suppl 3:S30
Choudhuri D, Huda T, Theodoratou E, Nair H, Zgaga L, Falconer R, Luksic I, Johnson HL, Zhang JS, El Arifeen S, Nelson CB, Borrow R, Campbell H, Rudan I	An evaluation of emerging vaccines for childhood meningococcal disease	BMC Public Health. 2011;11 Suppl 3:S29
Catto AG, Zgaga L, Theodoratou E, Huda T, Nair H, Arifeen SE, Rudan I, Duke T, Campbell H	An evaluation of oxygen systems for treatment of childhood pneumonia	BMC Public Health. 2011;11 Suppl 3:S28
Webster J, Theodoratou E, Nair H, Seong AC, Zgaga L, Huda T, Johnson HL, Madhi S, Rubens C, Zhang JS, El Arifeen S, Krause R, Jacobs TA, Brooks AW, Campbell H, Rudan I	An evaluation of emerging vaccines for childhood pneumococcal pneumonia	BMC Public Health. 2011;11 Suppl 3:S26
Yakoob MY, Theodoratou E, Jabeen A, Imdad A, Eisele TP, Ferguson J, Jhass A, Rudan I, Campbell H, Black RE, Bhutta ZA	Preventive zinc supplementation in developing countries: impact on mortality and morbidity due to diarrhea, pneumonia and malaria	BMC Public Health. 2011;11 Suppl 3:S23
Jackson SJ, Steer AC, Campbell H	Systematic review: Estimation of global burden of non–suppurative sequelae of upper respiratory tract infection: rheumatic fever and post–streptococcal glomerulonephritis	Trop Med Int Health. 2011;16: 2–11
Campbell A, Rudan I	A systematic review of birth cohort studies in Africa	J Global Health. 2011:1:46–58
McKinnon R, Campbell H	A systematic review of birth cohort studies in Asia	J Global Health. 2011:1:59–71
Waters D, Jawad I, Ahmad A, Lukšić I, Nair H, Zgaga L, Theodoratou E, Rudan I, Zaidi AKM, Campbell H	Aetiology of community–acquired neonatal sepsis in low– and middle–income countries	J Global Health. 2011; 1: 154–170
Velu PP, Gravett CA, Roberts TK, Wagner TA, Zhang JFS, Rubens CE, Gravett MG, Campbell H, Rudan I	Epidemiology and aetiology of maternal bacterial and viral infections in low– and middle–income countries	J Global Health 2011;1:171–88
Roberts T, Gravett CA, Velu PP, Theodoratou E, Wagner TA, Zhang JS, Campbell H, Rubens CE, Gravett MG, Rudan I	Epidemiology and aetiology of maternal parasitic infections in low– and middle–income countries	J Glob Health. 2011;1:189–200
Theodoratou E, Zhang JS, Kolcic I, Davis AM, Bhopal S, Nair H, Chan KY, Liu L, Johnson H, Rudan I, Campbell H	Estimating pneumonia deaths of post–neonatal children in countries of low or no death certification in 2008	PLoS One. 2011;6:e25095
Sivakumaran S, Agakov F, Theodoratou E, Prendergast JG, Zgaga L, Manolio T, Rudan I, McKeigue P, Wilson JF, Campbell H	Abundant pleiotropy in human complex diseases and traits	Am J Hum Genet. 2011;89:607–18
Edmond K, Scott S, Korczak V, Ward C, Theodoratou E, Clark A, Griffiths U, Rudan I, Campbell H	Global and regional estimates of disabling sequelae from pneumonia 1970–2011	PLoS One 2012;7:e31239
Baxter JM	One in a million, or one in thousand: What is the morbidity of rabies in India?	J Glob Health. 2012;2:010303
Herbert LJ, Middleton SI	An estimate of syphilis incidence in Eastern Europe	J Glob Health. 2012;2:010402
Jawad I, Lukšić I, Rafnsson SB	Assessing available information on the burden of sepsis: global estimates of incidence, prevalence and mortality	J Glob Health. 2012;2:010404
George–Carey R, Adeloye D, Chan KY, Paul A, Kolčić I, Campbell H, Rudan I	An estimate of the prevalence of dementia in Africa: A systematic analysis	J Glob Health. 2012;2:020401
Waters D, Theodoratou E, Campbell H, Rudan I, Chopra M	Optimizing community case management strategies to achieve equitable reduction of childhood pneumonia mortality: An application of Equitable Impact Sensitive Tool (EQUIST) in five low– and middle–income countries	J Glob Health. 2012;2:020402
Graham A, Adeloye D, Grant L, Theodoratou E, Campbell H	Estimating the incidence of colorectal cancer in Sub–Saharan Africa: A systematic analysis	J Glob Health. 2012;2:020404
Paul A, Adeloye D, George–Carey R, Kolčić I, Grant L, Chan KY	An estimate of the prevalence of epilepsy in Sub–Saharan Africa: A systematic analysis	J Glob Health. 2012;2:020405
Dowman B, Campbell RM, Zgaga L, Adeloye D, Chan KY	Estimating the burden of rheumatoid arthritis in Africa: A systematic analysis	J Glob Health. 2012;2:020406
Theodoratou E, Montazeri Z, Hawken S, Allum GC, Gong J, Tait V, Kirac I, Tazari M, Farrington SM, Demarsh A, Zgaga L, Landry D, Benson HE, Read SH, Rudan I, Tenesa A, Dunlop MG, Campbell H, Little J	Systematic meta-analyses and field synopsis of genetic association studies in colorectal cancer	J Natl Cancer Inst. 2012;104:1433–57
Nair H, Simőes EA, Rudan I, et al., Campbell H. for the Severe Pneumonia Working Group (including Hanlon P)	Global and regional burden of hospital admissions for severe acute lower respiratory infections in young children in 2010: a systematic analysis	Lancet. 2013;381:1380–90
Jackson S, Mathews KH, Pulanić D, Falconer R, Rudan I, Campbell H, Nair H	Risk factors for severe acute lower respiratory infections in children: a systematic review and meta–analysis	Croat Med J. 2013;54:110–21
Luksic I, Kearns PK, Scott F, Rudan I, Campbell H, Nair H	Viral aetiology of hospitalized acute lower respiratory infections in children under 5 years of age	Croat Med J. 2013;54:122 –34
Luksic I, Clay S, Falconer R, Pulanić D, Rudan I, Campbell H, Nair H	Effectiveness of seasonal influenza vaccines in children: a systematic review	Croat Med J. 2013;54:135–45
Kokki I, Papana A, Campbell H, Theodoratou E	Estimating the incidence of colorectal cancer in South East Asia	Croat Med J. 2013;54:532–40
Luksic I, Mulic R, Falconer R, Orban M, Sidhu S, Rudan I	Estimating global and regional morbidity from acute bacterial meningitis in children: assessment of the evidence	Croat Med J. 2013;54:510–8
Nair H, Lau ESM, Brooks WA, Seong AC, Theodoratou E, Zgaga L, Huda T, Jadhav SS, Rudan I, Campbell H	An evaluation of the emerging cross–protective vaccines against influenza in children	BMC Public Health. 2013:13 (Suppl 3):S14
Bhopal A, Callender T, Knox AF, Regmi S	Strength in numbers? Grouping, fund allocation and coordination amongst the neglected tropical diseases	J Glob Health. 2013;3:020302
Geldsetzer P, Williams TC, Kirolos A, Mitchell S, Ratcliffe LA, Kohli–Lynch MK, Bischoff EJ, Cameron S, Campbell H	The recognition of and care seeking behaviour for childhood illness in developing countries: a systematic review	PLoS One. 2014; 9:e93427
Cheema A, Adeloye D, Sidhu S, Sridhar D, Chan KY	Urbanization and prevalence of type 2 diabetes in Southern Asia: A systematic analysis	J Glob Health. 2014;4:010404
Lo A, Polsek D, Sidhu S	Estimating the burden of neural tube defects in low– and middle–income countries	J Glob Health. 2014;4:010402
Adeloye D, Basquill C	Estimating the prevalence and awareness rates of hypertension in Africa: a systematic analysis	PLoS One. 2014;9:e104300
Adeloye D, Basquill C, Papana A, Chan KY, Rudan I, Campbell H	An estimate of the prevalence of COPD in Africa: a systematic analysis	COPD. 2015;12:71–81
Adeloye D, Basquill C, Aderemi AV, Thompson JY, Obi FA	An estimate of the prevalence of hypertension in Nigeria: a systematic review and meta–analysis	J Hyperten. 2015;33:230–42
Kennedy ED, Fairfield CJ, Fergusson SJ	A neglected priority? The importance of surgery in tackling global health inequalities	J Glob Health. 2015;5:020304
Rudan I, Sidhu S, Papana A, Meng SJ, Xin–Wei Y, Wang W, Campbell–Page RM, Demaio AR, Nair H, Sridhar D, Theodoratou E, Dowman B, Adeloye D, Majeed A, Car J, Campbell H, Wang W, Chan KY	Prevalence of rheumatoid arthritis in low and middle income countries: A systematic review and analysis	J Glob Health. 2015;5:010409
Chan KY, Yu XW, Lu JP, Demaio AR, Bowman K, Theodoratou E	Causes of accidental childhood deaths in China in 2010: A systematic review and analysis	J Glob Health. 2015;5:010409
Ting Shi, McLean K, Campbell H, Nair H	The etiological role of common respiratory viruses in acute lower respiratory infections in children under five years: A systematic review and meta–analysis	J Glob Health. 2015;5:020408

**Figure:**
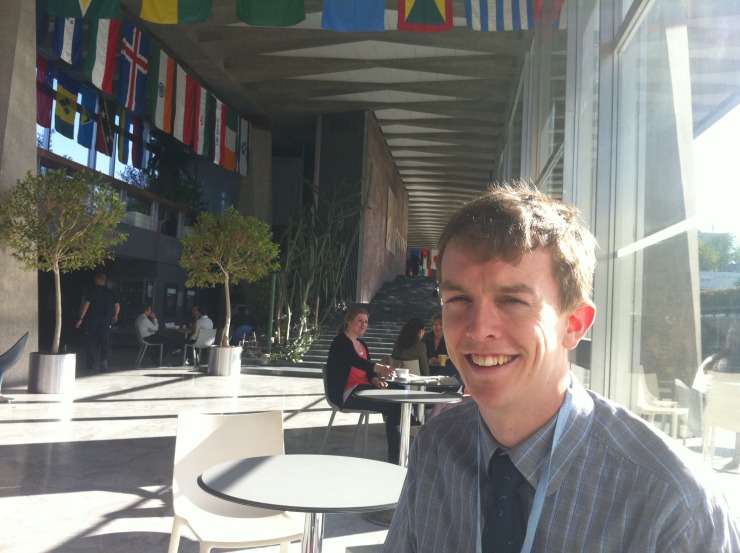
Photo: EUGHS member Donald Waters visited the World Health Organization to present the results of his research on the etiology of newborn infections.

**Figure:**
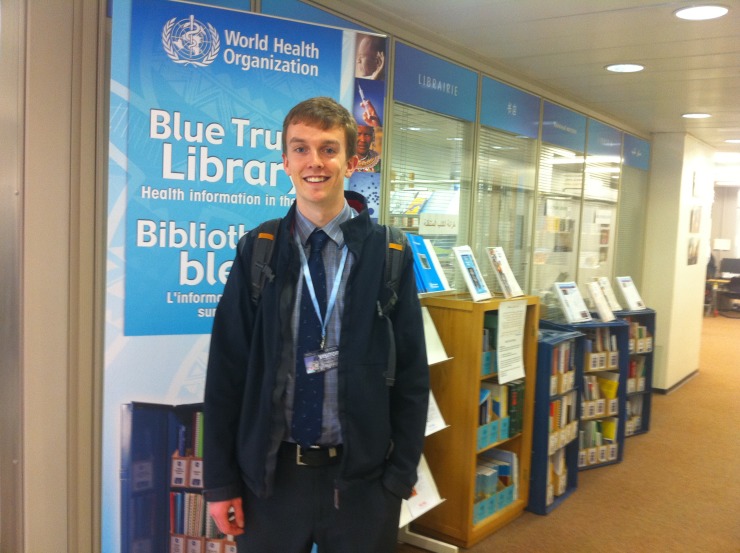
Photo: EUGHS member Donald Waters in the library of the World Health Organization.

**Figure:**
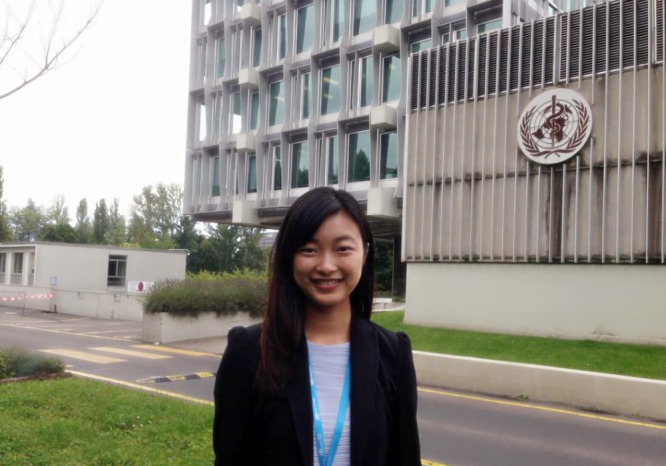
Photo: EUGHS member Katy Wong spent several months at the World Health Organization in Geneva as an intern.

## EUGHS student presentations at international meetings

EUGHS students had several successful presentations of their research (**Table 2**) and internships at the WHO (**Box 1**).

**Table 2:** EUGHS student presentations at meetings

Student	Meeting/topic
Andrew McCallum	Meeting with staff of the Department for Child and Adolescent Health and Development, World Health Organization, Geneva, Switzerland, May 2006 (student presentation on role of medical schools in assembling evidence and their role in the International Child Health Review Collaboration)
Amy Agahi Victoria Anderson Issrah Jawad Rachel Falconer	Meeting of the World Health Organization's Child Health Epidemiology Reference Group (CHERG): Dubrovnik, Croatia, September 2008 (*student presentations of systematic reviews of global burden of disease for a variety of childhood infections)*
Rachel Falconer Issrah Jawad Sarah Ali Sameeka Kariyawasam Sue Johnson Andy Davis Felicity Woodward Joy Ferguson Jasjot Singhota Neil Gray	Meeting of the World Health Organization's Child Health Epidemiology Reference Group (CHERG): Dubrovnik, Croatia, September 2009 (*student presentations of systematic reviews of global burden of disease for a variety of childhood infections and reviews of effectiveness of interventions against these conditions)*
Debajeet Choudhuri Arnoupe Jhass Vas Verma Joy Ferguson Daisy Higginson Ang Choon Seong Alasdair Catto Julia Webster	Meeting of the Bill and Melinda Gates Foundation's Advisory group on research priorities for emerging interventions against childhood pneumonia, meningitis and influenza: Dubrovnik, Croatia, September 2009 (*student presentations of systematic reviews of emerging interventions against childhood pneumonia, meningitis and influenza)*
Anna Breakey	Meeting of the World Health Organization's Child Health Epidemiology Reference Group (CHERG): Tarrytown, New York, USA, October 2009 (*student presentation of systematic reviews of global burden of disease for childhood infections)*
Peter Hanlon	Meeting of the World Health Organization's Child Health Epidemiology Reference Group (CHERG) on the global burden of severe pneumonia in young children: Edinburgh, UK, September 2010 (*student presentation of systematic review on severe pneumonia to open meeting)*
Alasdair Campbell	World Health Organization's meeting to review recommendations on newborn health, childhood diarrhoea and pneumonia: Geneva, Switzerland, February 2011 *(international expert group meeting to undertake GRADE assessment of WHO recommendations for pocketbook of hospital care for children – student presentation on role of medical schools in assembling evidence)*
Prasad Palani Velu Tom K. Roberts	International conference on neonatal sepsis: New Dehli, India, March 2011(*student presentation of systematic reviews of global burden of disease for maternal bacterial, viral and parasitic infections and newborn infections)*
Donald Waters	World Health Organization's meeting on equitable investment in child health interventions and EQUIST tool: Geneva, Switzerland, April 2011 (*student presentation of application of EQUIST tool on childhood pneumonia in 5 countries)*
Donald Waters	UNICEF's meeting on equitable investment in child health interventions and EQUIST tool: Innocenti Centre, Florence, Italy, October 2012 (*student presentation of application of EQUIST tool on childhood pneumonia in 5 countries)*
Donald Waters Thomas Williams Kenneth McLean	Meeting of the World Health Organization's Child Health Epidemiology Reference Group (CHERG) / Maternal and Childhood Epidemiology Estimates (MCEE) on the global child mortality causes: Dubrovnik, Croatia, July 2014 (*student presentation of systematic reviews of global burden of disease for maternal and childhood infections)*
Anna Badenhorst	Meeting of the Child Health and Nutrition Research Initiative (CHNRI) and Global Coalition of Child Health Centres (GCCHC) on research priorities for global child health: Toronto, Canada, October 2014 (*student presentation of global, regional and national capacity to conduct global health research between 1995 and 2010)*

## My personal experience as WHO intern – by Rachel Burge

In the summer of 2014, I was delighted to have the opportunity to undertake an internship at the World Health Organisation Headquarters in Geneva, Switzerland, in the Department of Maternal, Child and Adolescent Health. I was a medical student having just finished my third year of study at Edinburgh University, graduating with an intercalated degree in Infectious Disease and before returning to the third year medical curriculum. Up until that time, I had taken advantage of the many opportunities offered within the university to build my knowledge and understanding of global public health, developing an interest in world current affairs and learning how I may be able to focus my medical career in a globally–minded direction.

**Figure:**
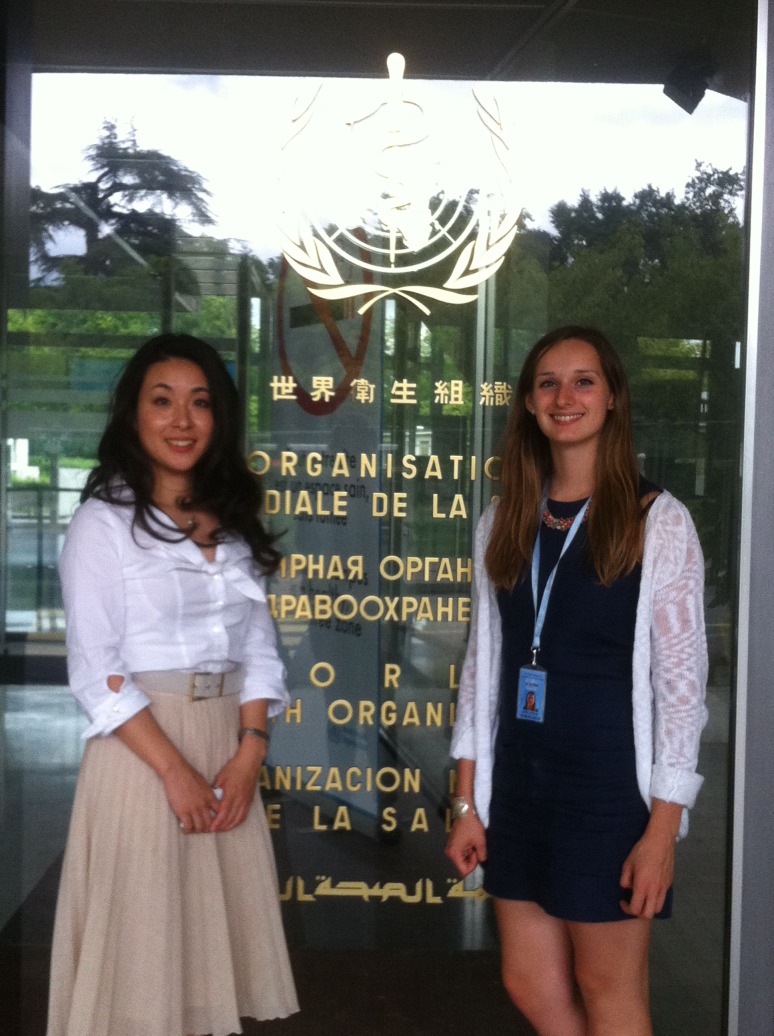
Photo: EUGHS member Rachel Burge (right) with her host at the World Health Organization, Dr Sachiyo Yoshida (left).

I had been involved with the University of Edinburgh Global Health Society since commencing university, and it was through the society and the university staff involved that the internship was organised – for which I will always be so grateful! For me, the opportunity of an internship at the World Health Organisation, an organisation so central to so many current and historical medical global affairs which were so prominent in discussion was an opportunity too good to surpass. Indeed, even despite the big expectations of a passionate young medical student, my time in Geneva still exceeded expectations. The opportunities to meet the people who worked with and in collaboration with the organisation, to ask questions and hear differing views and advice, to get to know how they journeyed into the world of public health, and to get a glimpse into the workings of this huge organisation was invaluable to me. Being able to contribute to this work, although in a small way, was of course an extremely exciting prospect.

For my six week internship, I was given set tasks by my supervisor, Sachiyo Yoshida, a technical officer in the department. Reflecting her own work, my tasks focused more specifically on neonatal and child health. A large portion of my workload focused on the Child Health Nutrition and Research Initiative, which published a methodology in 2009 which aimed to rank research priorities in order to guide research scientists and their funders. In this sense, my work was the beginning of a retrospective study looking at the research which targeted the top 5 research priorities as ranked by the initiative, concerned with reducing the top 5 causes of child mortality. I searched the literature for research published since 2011 up until 2014. Of the relevant studies identified, I then went on to identify their funders, information which could ultimately be used to evaluate the impact of the CHNRI publication.

Aside from this main study, I was able to contribute to the write–up of a Study Protocol, and to the preparations for World Breastfeeding Week – promoting conference within the headquarters, attending conferences and taking notes for the panel. I enjoyed meeting a speaking with globally–minded people, who had devoted their career to global health, and often had fantastic stories to be told. Aside from my work at WHO, I also met interns working elsewhere in the HQ, and elsewhere in Geneva for other United Nations agencies, spending time getting to know the beautiful city in the summer months with like–minded young aspiring global health geeks.

**Figure:**
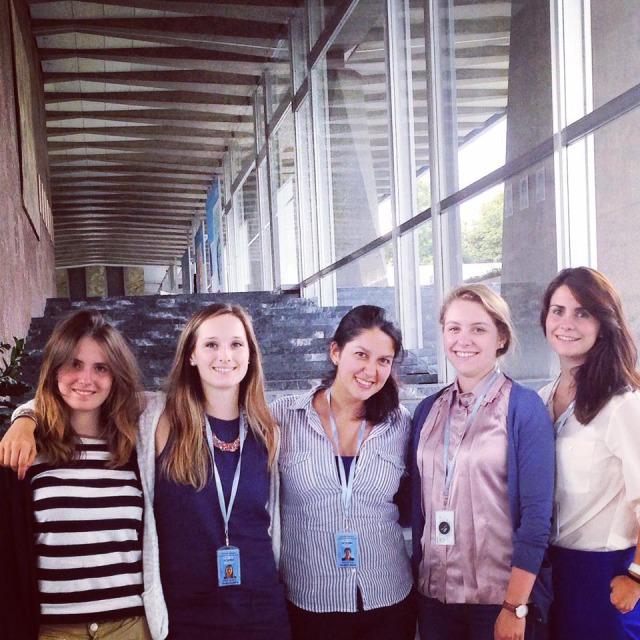
Photo: EUGHS member Rachel Burge (second from the left) with other interns at the World Health Organization in Geneva.

Once the weeks had so quickly passed and my internship came to an end, I left inspired by both the people I met and the work I had done during my stay in Geneva. I must give special thanks to the University’s Global Health Society and the staff involved for arranging the internship, and to the Innovative Initiative Grant at the University of Edinburgh for providing funding, without which I would not have been able to take this opportunity. I have every intention to continue the development of my involvement with global health and to build upon the foundations for a medical career focused in the direction of global health, and I am sure that my experience at the WHO will influence my interests and choices throughout my future career.

## My personal experience as WHO intern – by Katy Nuen–wing WONG

Upon graduating from the master of public health programme at the University of Edinburgh, I aspired to have a practical experience in public health to consolidate what I had learned throughout the course. Doing an internship at the World Health Organization (WHO) was undeniably a great opportunity to learn the global progress on the enhancement of population health. Therefore, I submitted my application to the WHO website, indicating my great interest in certain projects. Thankfully, I heard from the WHO after a few months of application, and was successfully offered an internship opportunity after a phone interview. Having the background as a registered Chinese medicine practitioner in Hong Kong, I was assigned to the Team of Traditional and Complementary Medicine at the WHO headquarters in Geneva. There, I spent an unforgettable experience of five months.

**Figure:**
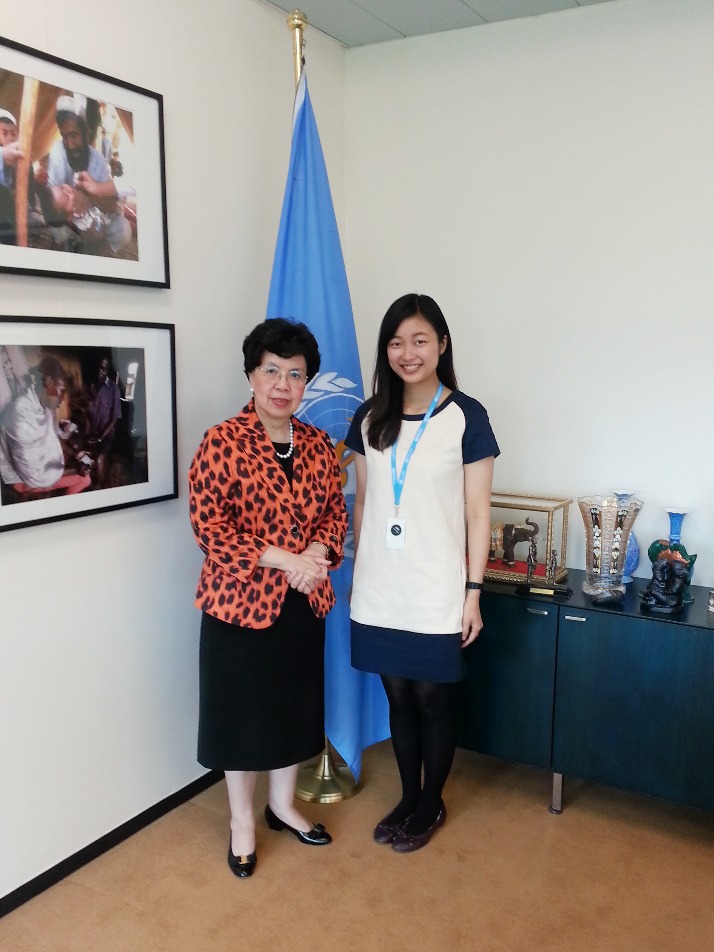
Photo: EUGHS member Katy Wong (right) with the Director–General of the World Health Organization, Dr Margaret Chan (left).

WHO is the United Nation’s leading authority on international public health. Working closely with public health experts around the world and tackling different important health issues was a very challenging and exciting task. It also gave me great motivation to go to work knowing that it would exert great influence to many populations which could directly enhance their health. My main task in the internship was to work with traditional medicine experts on data verification and analysis on the WHO global surveys. Thanks to the epidemiology and statistics training that I received at the University of Edinburgh, I was well–prepared to manage and analyze the survey data obtained from the 193 member states of the WHO. During my internship I was also involved in working on the unprecedented development of the International Classification of Traditional Medicine which would be included in the coming ICD–11. This achievement will make significant contribution to the standardization of the clinical language used by traditional medicine to facilitate information exchange and the integration of complementary medicine into the health care system. All the work I conducted there was evaluated by my supervisors at WHO, whom eventually offered me an exceptional extension of the 3–month internship contract on top of the first contract.

On the other hand, as an intern I got to participate in many training sessions and discussion seminars which helped expand and develop my public health knowledge. I had attended seminars on mental health, palliative care and neglected tropical disease. I also had the privilege of being present at the World Peace Talk of the United Nations to learn the issues regarding world peace and human right. Coincidently, it was the Ebola outbreak period during the internship, hence, I experienced firsthand the outbreak response, leadership, roadmap development, division of labour, press conferences, and even the sharing from experts who were deployed in the field in West Africa. Attending the Ebola meeting with the Director–General of the WHO, Dr Margaret Chan, and the Secretary–General of the United Nations, Dr Ban Ki–moon, on the discussion of Ebola vaccine and treatment was very impressive. I also joined the communication team to contact Ebola experts worldwide to investigate the possible treatments for Ebola.

**Figure:**
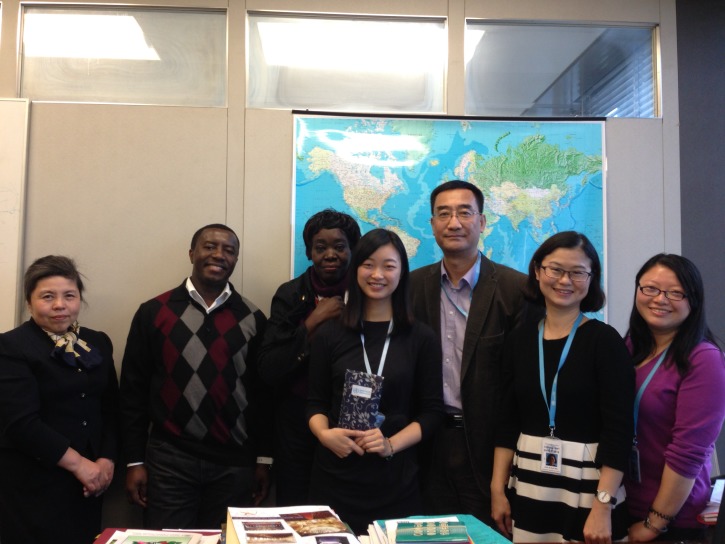
Photo: EUGHS member Katy Wong (in the middle) with the Team of Traditional/Complementary Medicine at the World Health Organization in Geneva.

Another memorable experience was the duty travel to Macao SAR, China, for a WHO training workshop. Working as WHO secretariat, I learned the administration and logistics in organizing an international event. In the training workshop, I got the opportunity to meet with a lot of government officials from the ministries of health of different countries. I had gained great insight from the country leaders on policy–making and international cooperation in consumer protection. Together we had built beautiful memories and friendships.

Apart from the tasks that I performed, I met so many amazing and interesting people from all over the world. I enjoyed very much the working environment at the WHO headquarters in which I worked closely with colleagues with multi–cultural and multi–disciplined backgrounds, which can lead to many brilliant ideas. I had also joined the WHO intern board as a interns coordinator, where I made friends with many amazing interns who will possibly become the public health leaders in the future. During our coffee time and excursions, we discussed a lot on the infectious disease control measures, the development of medicines and the research methodology. It was indeed an excellent platform for young people who share the same aspiration to meet and work towards our goals.

Above all, this internship has exposed me to different global health issues, and allowed me to view traditional medicine from an international perspective. Now I understand a lot more the pathway to develop traditional medicine, in terms of quality and safety, pharmacovigilance capacity, national policies and regulations. It has greatly broadened my horizon to understand health issues in a global context. This internship is a once–in–a–lifetime experience for me. It is definitely something that I would highly recommend for everyone who is interested in public health to apply for.

Box 1 **Internships at the World Health Organization for EUGHS students**

• Rachel Burge [2014] – 6 weeks (WHO Headquarters, Geneva, Switzerland)

• Katy Wong [2014] – 5 months (WHO Headquarters, Geneva, Switzerland)

• Mia Cokljat [2015] – 6 weeks (WHO Headquarters, Geneva, Switzerland)

• Kirstie–Ann McPherson [2015] – 6 weeks (WHO Headquarters, Geneva, Switzerland)

• James Gao [2015] – 6 weeks (WHO Headquarters, Geneva, Switzerland)

• Kenneth McLean [2015] – 6 weeks (WHO Headquarters, Geneva, Switzerland)

